# MyD88 Associated ROS Generation Is Crucial for *Lactobacillus* Induced IL-12 Production in Macrophage

**DOI:** 10.1371/journal.pone.0035880

**Published:** 2012-04-20

**Authors:** Shintaro Ichikawa, Mika Miyake, Rei Fujii, Yutaka Konishi

**Affiliations:** Central Laboratories for Frontier Technology, Kirin Holdings Co., Ltd., Yokohama, Japan; Indian Institute of Science, India

## Abstract

It is well known that some strains of lactic acid bacteria (LAB) can induce IL-12 which plays an important role in modulating immune responses. However, the mechanisms by which LAB induce IL-12 production remain unclear. Here, we examine the role of toll-like receptors (TLR's) and reactive oxygen species (ROS) in IL-12 production by LAB stimulated peritoneal macrophages. Our results indicate that a TLR is not necessary for IL-12 induction by LAB, whilst the universal adaptor protein, MyD88, is essential. Specific strains of LAB induced ROS that correlated with both the frequency of phagocytosis and IL-12 production. Reduction in IL-12 production by NADPH oxidase inhibitors or ROS scavengers demonstrates the crucial role of ROS in IL-12 induction. Interestingly, deficiency of TLR2, 4, 9 or MyD88 did not affect the phagocytosis of LAB strain KW3110, a potent IL-12 inducer, and ROS production was significantly reduced only in MyD88 deficient macrophages. These results suggest the existence of TLR-MyD88 independent LAB recognition and MyD88 related ROS induction mechanisms. We show here the importance of ROS for IL-12 induction and provide new insights into IL-12 induction by LAB.

## Introduction

Lactobacilli are one of many commensal microbes and are known to play important roles in maintaining the health of their host [1]. Recently lactic acid bacteria (LAB) have become popular as probiotics i.e. living microorganisms that confer health benefits [2], [3]. Among the more beneficial roles of lactobacilli, is their influence on immunological function. It is known that lactobacilli can have anti-tumor, anti-infectious and anti-allergic functions [4]–[8]. Although the mechanisms by which lactobacilli exhibit these functions are not fully understood, one possible key factor is IL-12. IL-12 is an important cytokine which induces T helper (Th) 1 type immune responses and enhances cellular immunity. Some specific LAB strains are known to induce IL-12 production [9]–[11]. Recently, it has been shown that IL-12 is important in LAB mediated natural killer cell activation [12], [13]. The anti-allergic effects of specific LAB strains may also be explained by their IL-12 induction [10], [14], [15]. We have shown that *Lactobacillus paracasei* strain KW3110 (KW) induces IL-12 and suppresses allergic symptoms in an atopic dermatitis model [10], [16]. Although a number of reports have suggested IL-12 is important for LAB mediated immune modulation, it is still unclear why and how specific LAB induce IL-12 production.

Toll-like receptors (TLRs) comprise a family of receptors that recognize specific molecular patterns related to microbial components [17], [18]. TLR2 and TLR4 are required for the recognition of bacterial cell wall components such as lipoprotein and lipopolysaccharide (LPS) [19] whereas TLR7 and TLR9 are receptors for bacterial internal components such as single stranded RNA and CpG-DNA [20], [21]. These receptors act as the first line of defense and play an important role in the innate immune system. Interaction of TLRs and their ligands leads to the activation of signaling cascades through the adaptor molecule myeloid differentiation factor 88 (MyD88), followed by cytokine production, including IL-12 [22]. Several studies have suggested that peptidoglycan or CpG ODN, derived from LAB, induces IL-12 production via TLR recognition [23], [24]. However, even with enriched LAB components, IL-12 induction is weaker than that of intact LAB [25]. In addition, we have previously suggested that intact LAB strains can induce IL-12 production independently of TLR2, 4 and 9, although MyD88 is still necessary [26]. Thus it is unlikely that TLRs are essential for IL-12 induction by LAB.

It is well documented that phagocytosis is an important mechanism in innate immune defense [27]. Microbes are detected by various phagocytic receptors e.g. Fc-receptors, complement receptors or mannose receptors [28]–[30]. TLRs do not induce phagocytosis and are not categorized as phagocytic receptors. Phagocyte-microbe contact is accompanied by internalization of microbes, activation of intracellular signaling, microbial killing mechanisms and production of pro- and anti-inflammatory cytokines [27]. Among the various responses following phagocytosis, production of reactive oxygen species (ROS) by NADPH oxidase is the most critical factor for removal of microbes [31], [32]. Phagocytic signaling followed by ROS production is also important for IL-12 induction [33], [34].

In this study, we investigate the role of ROS in LAB induced IL-12 production by peritoneal macrophages and reveal that ROS induction, accompanied by LAB phagocytosis, is necessary for IL-12 secretion. We also find that MyD88 is important, not for TLR mediated LAB recognition, but for ROS induction after LAB phagocytosis.

## Results

### IL-12 production correlates with the frequency of LAB phagocytosis and NADPH oxidase mediated ROS production

**Figure 1:**
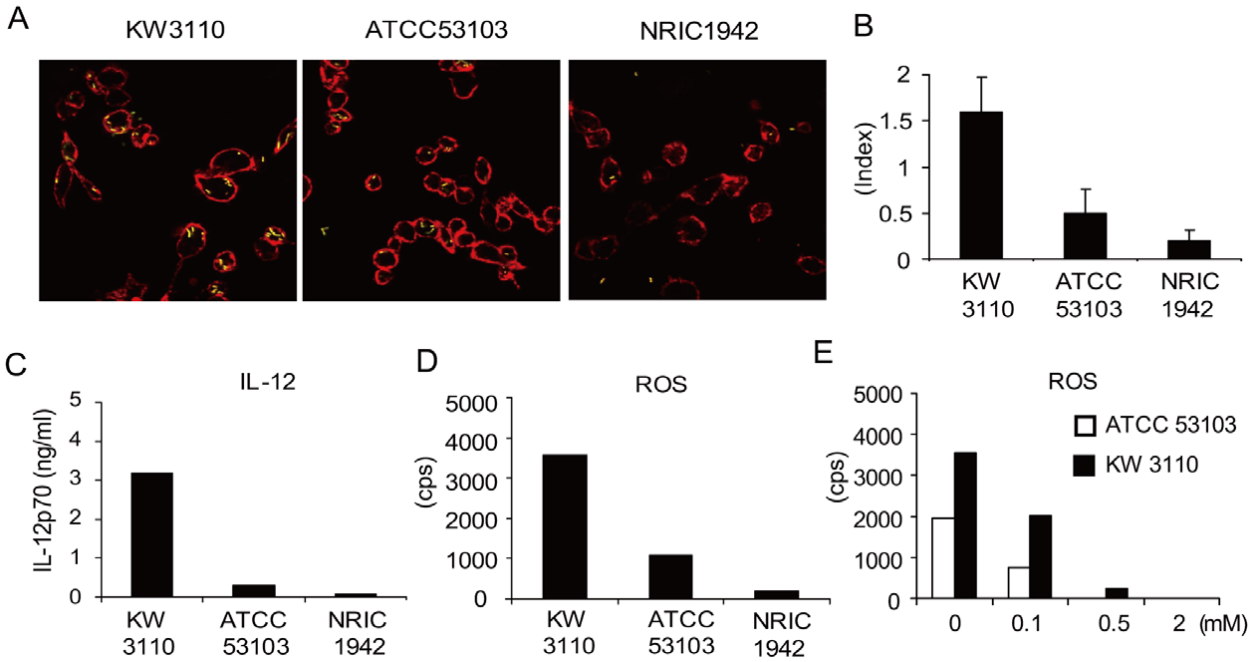
IL-12 production by macrophage correlates with the frequency of LAB phagocytosis and ROS production. (A, B) Peritoneal macrophages were cultured with FITC-labeled identical LAB strains (3 µg/ml) in a chamber slide. Following the culture for 8 hrs, macrophages were stained with CD11b antibody (red) and phagocytosis of LAB (green) was analyzed by confocal microscopy. Representative pictures (A) and mean phagocytosis index ± SD (B) are shown. (C) Peritoneal macrophages were cultured with 1 µg/ml LAB strains for 24 hr and the levels of IL-12p70 in the culture supernatants were determined by ELISA. (D) Peritoneal macrophages were cultured with 1 µg/ml KW3110, ATCC53103 or NRIC1942 for 8 hrs. ROS were measured using the luminescent dye L-012 at the end of culture. (E) Peritoneal macrophages were cultured with KW3110 (filled) or ATCC53103 (blank) with apocynin (0, 0.1, 0.5, 2 mM) and ROS production measured similarly. Values are average of duplicated culture except figure 1B. Representative data from more than three independent experiments yielding consistent results are shown.

We used peritoneal macrophages for our experimental system, because previous report suggested that it is not DCs but macrophages which mainly produce IL-12 by LAB stimulation [35]. Since bacteria phagocytosis by macrophages is a central event in the induction of immunological responses, we examined whether the frequency of LAB phagocytosis is related to IL-12 production using three different LAB strains: KW3110, ATCC53103 and NRIC1942. FITC-labeled strains were cultured with peritoneal macrophage and LAB phagocytosis was analyzed by confocal microscopy (Fig. 1A). The frequency of LAB intake is represented by the phagocytosis index (Fig. 1B). When we measured IL-12 production by macrophage stimulated using the same strains, the amount correlated with the phagocytosis index (Fig. 1C).

An oxidative burst is induced in macrophages after the phagocytosis of bacteria. We examined reactive oxygen species (ROS) production by macrophages after LAB stimulation. ROS production was observed 8 hrs after LAB stimulation and clearly correlated with IL-12 production and the phagocytosis index (Fig. 1D). We then used different LAB strains in the culture to gather more general information. There was significant correlation (*r* = 0.67, *p*<0.001) between IL-12 and ROS production in LAB-stimulated macrophages (Table 1). ROS production was dose dependently reduced by apocynin, a NADPH oxidase specific inhibitor (Fig. 1E), suggesting that phagocytosed LAB induces ROS *via* NADPH oxidase activation.

**Table 1:** IL-12 and ROS production from macrophages stimulated by various strains of LAB.

*genus/species*	strain	IL-12 (pg/ml)	ROS (cpm)
*Lactobacillus paracasei*	KW3110	3187	3568
*Lactobacillus paracasei*	JCM1133	372	546
*Lactobacillus paracasei*	NRIC1936	425	537
*Lactobacillus paracasei*	NRIC1942	80	203
*Lactobacillus paracasei*	NRIC1946	583	295
*Lactobacillus paracasei*	JCM1181	1515	2270
*Lactobacillus paracasei*	JCM1111	352	539
*Lactobacillus paracasei*	IFO12004	555	618
*Lactobacillus casei*	LB81	1316	2247
*Lactobacillus brevis*	IFO3345	191	768
*Lactobacillus brevis*	JCM1059	966	2352
*Lactobacillus brevis*	L8	202	1193
*Lactobacillus brevis*	L42	391	2910
*Lactobacillus brevis*	L57	0	347
*Lactobacillus brevis*	L63	243	931
*Lactobacillus brevis*	L107	0	369
*Lactobacillus brevis*	TUML43	0	594
*Lactobacillus brevis*	JCM1170	258	2569
*Lactobacillus helveticus*	B-1	108	1813
*Lactobacillus helveticus*	JCM1120	0	839
*Lactobacillus acidophilus*	L54-1	687	1902
*Lactobacillus gasseri*	JCM1131	1156	1278
*Lactobacillus plantarum*	JCM1149	2431	2538
*Lactobacillus jonsonii*	JCM2012	905	2902

Macrophages were cultured with each LAB for 8 and 24 hours for measurement of ROS and IL-12 respectively. (*r* = 0.68, *p*<0.001).

### Kinetic analysis of ROS production, IL-12 mRNA expression and IL-12 production after LAB stimulation

**Figure 2:**
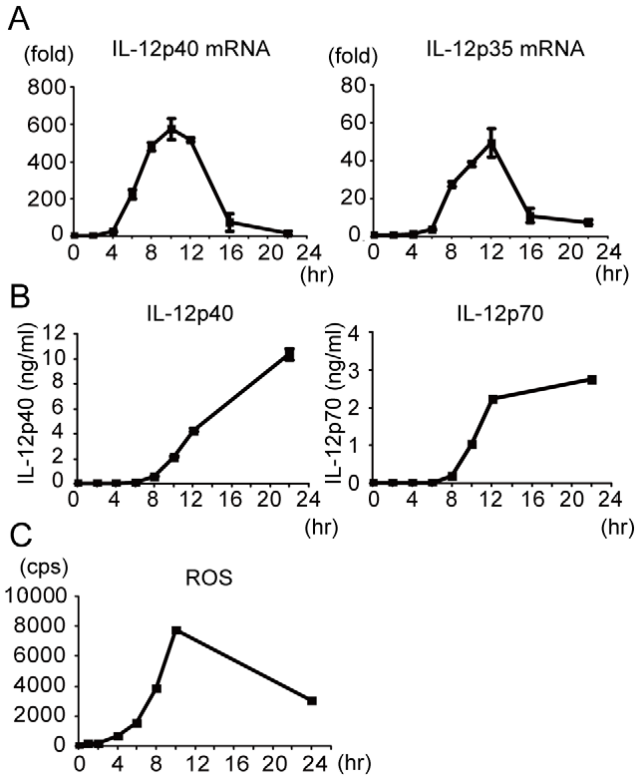
Kinetic analysis of ROS production, IL-12 mRNA expression and IL-12 production from macrophage after LAB stimulation. Peritoneal macrophages were cultured with 1 µg/ml KW3110 and IL-12p35 and IL-12p40 mRNA expression (A), IL-12p40 and IL-12p70 production (B) and ROS production (C) were measured over time. Values are mean of duplicate culture (C) or mean ± SD of triplicate culture (A, B). Data are representative of three independent experiments yielding similar results.

Next we examined the kinetic profiles of IL-12 mRNA expression, IL-12 production and the ROS production of macrophage stimulated by LAB strain KW3110 over 24 hrs. There are few reports on these profiles recorded in the literature. Increases of IL-12p40 and p35 mRNA expression began 4 hrs after stimulation (Fig. 2A). IL-12p40 and p35 mRNA expression expanded after 6 hr and 8 hr respectively. Following IL-12 mRNA expression, we detected IL-12p40 and p70 proteins in the cell culture medium 8 hrs after stimulation (Fig. 2B). However, ROS production started 2 hr after stimulation, i.e. earlier than IL-12p35 and p40 mRNA expression. ROS production continued for at least 10 hr (Fig. 2C). These kinetic analyses reveal that ROS production after LAB stimulation is an earlier event than IL-12 mRNA expression in macrophage.

### ROS is necessary for IL-12 production of LAB stimulated macrophage

**Figure 3:**
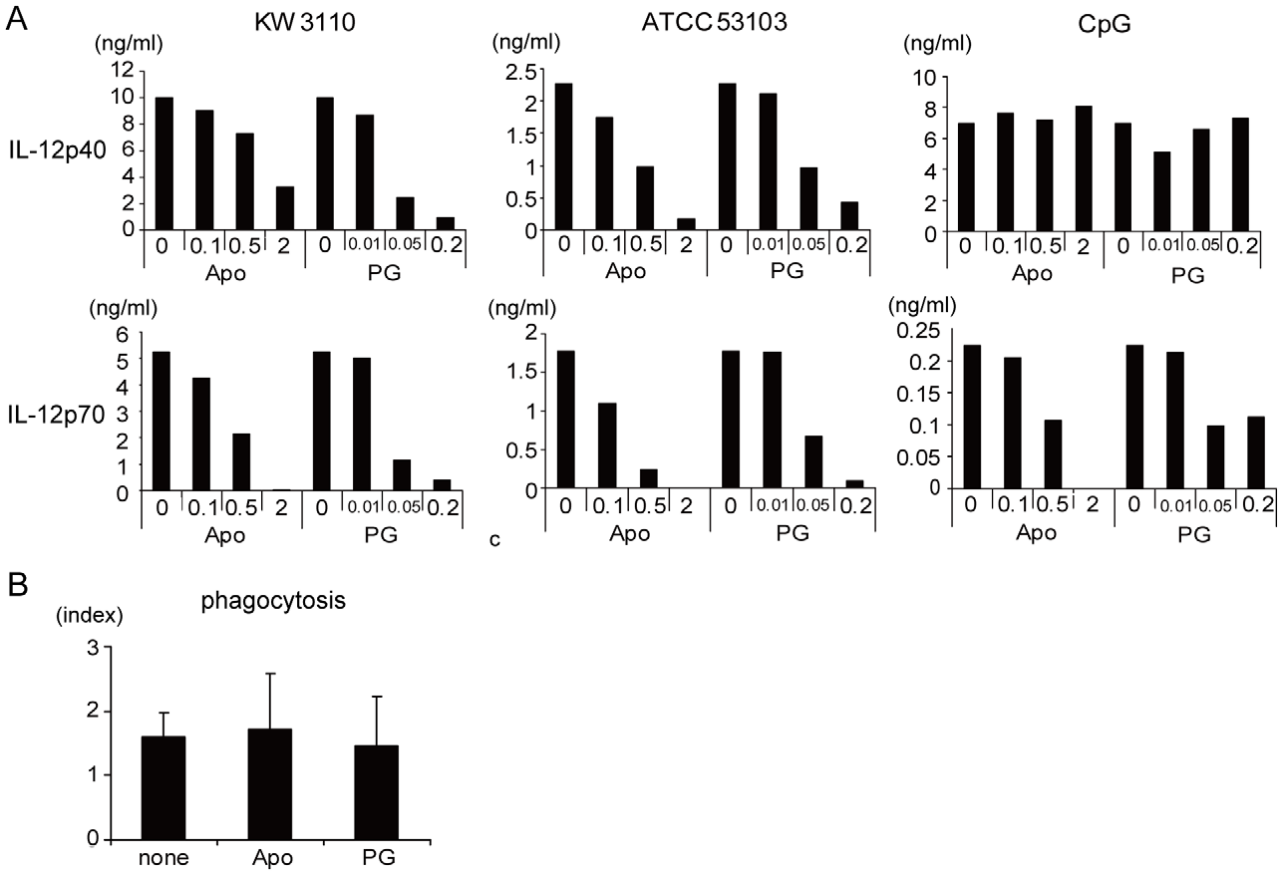
ROS dependent IL-12 production from LAB stimulated macrophage. (A) Peritoneal macrophages were cultured with 1 µg/ml KW3110, ATCC53103 or 1 µM CpG for 24 hrs in the presence of 0, 0.1, 0.5 or 2 mM apocynin (Apo) or 0, 0.01, 0.05 or 0.2 mM propyl gallate (PG). IL-12p40 and IL-12p70 in the cell culture supernatant were measured by ELISA. (C) Peritoneal macrophages were cultured with FITC-labeled identical KW3110 (3 µg/ml) in the presence of Apocynin or propyl gallate. Phagocytosis index was calculated in the same manner as described in Fig. 1A. Data are representative of three independent experiments.

The fact that ROS production is earlier than IL-12 mRNA expression supports the idea that ROS is the key factor for IL-12 induction by LAB stimulated macrophages. To investigate the IL-12 requirement for ROS, NADPH oxidase inhibitor (apocynin) and ROS scavenger (propyl gallate) were added to the cell culture medium. Apocynin or propyl gallate decreased IL-12p40 and IL-12p70 production after KW3110 or ATCC53103 stimulation (Fig. 3A). Interestingly, neither apocynin nor propyl gallate inhibit IL-12p40 production in CpG activated macrophage. It suggests that these inhibitors do not directly inhibit IL-12p40 production or kill macrophages. Importantly, apocynin and propyl gallate did not affect LAB phagocytosis (Fig. 3B, Dunnett's test).

### MyD88 dependent but TLR independent IL-12 production from peritoneal macrophages stimulated by LAB

**Figure 4:**
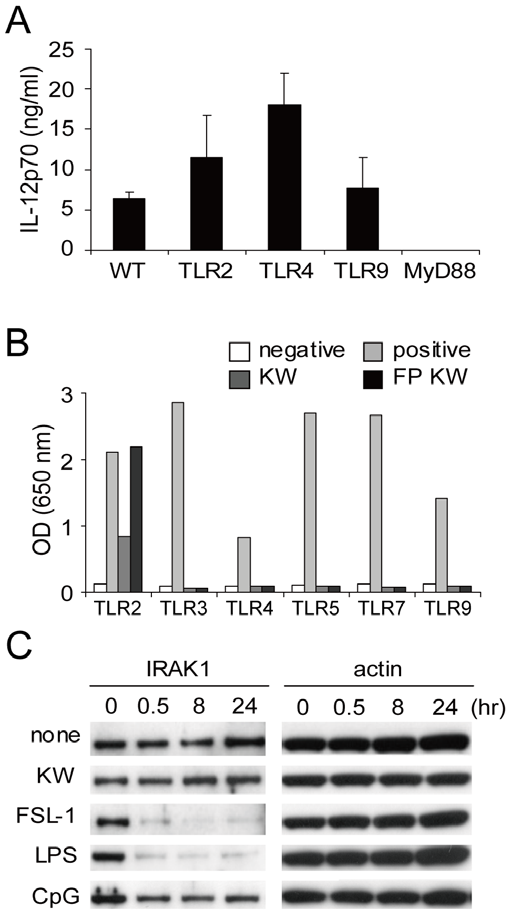
MyD88 dependent but TLR independent IL-12 production from peritoneal macrophages stimulated by LAB. (A) Peritoneal macrophages from wild type or TLR2-, 4-, 9- or MyD88-deficient mice were cultured with 1 µg/ml KW3110 for 24 hrs. IL-12p70 production was determined by ELISA. Data value is the mean ± SD of three mice. (B) KW3110, French pressed KW3110 or positive ligand for each TLRs were cultured with HEK 293 cells expressing indicated TLR. TLR stimulation was analyzed by assessing alkaline phosphatase activity (OD650 nm) which is under the control NF-κB activation. Data are representative of two independent experiment yielding same results. (C) Peritoneal macrophages from wild type mice were cultured with 1 µg/ml KW3110, FSL-1, LPS or 1 µM CpG. Cells were recovered at the indicated time points (0, 0.5, 8, 24 hr) and analyzed by Western blot for IRAK-1 and actin. Data are representative of two independent experiments.

We have previously shown that LAB stimulated, bone marrow-derived, dendritic cells and splenocytes produce IL-12 via a MyD88 dependent mechanism but do not require TLR2, 4 or 9 [26]. To confirm this observation we used peritoneal macrophages, from wild-type (WT) and MyD88-, TLR2-, 4- and 9- deficient mice were cultured for measurement of IL-12 production. Macrophages from TLR2-, 4- and 9-deficient mice produced similar or increased amounts of IL-12 relative to macrophages from WT mice, while MyD88-deficient macrophage did not produce IL-12 at all (Fig. 4A). It is true that the LAB strain may have more than two TLR ligands and can thus compensate for TLR deficiency (TLR2, 4 or 9). We therefore tested whether the LAB strain has multiple TLR ligands by assessing NF-κB activation in HEK293 cells expressing a given TLR (2, 3, 4, 5, 7, 9). We used intact KW3110 and French pressed KW3110 to make intracellular components accessible to HEK293 cells. It was apparent that only KW3110 have TLR2 ligands among those we tested (Fig. 4B).

We then investigated the activation of the IRAK-1 molecule that binds to MyD88 and mediates signal transduction [18]. Peritoneal macrophages were stimulated with KW3110 or TLR ligand (FSL-1, LPS and CpG) for 0, 0.5, 8, and 24 hrs and IRAK-1 activation detected by Western blotting. Activated IRAK-1 is digested by proteases following its phosphorylation [36], [37] and, in response to FSL-1, LPS or CpG, we observed a decrease in IRAK-1 as a consequence of degradation by these proteases [36], [37] (Fig. 4C). However, the protein band did not decrease when macrophages were stimulated by KW3110. This result suggests that KW3110 stimulation does not activate the TLR signaling pathway.

### MyD88-deficient macrophages show impaired ROS production

**Figure 5:**
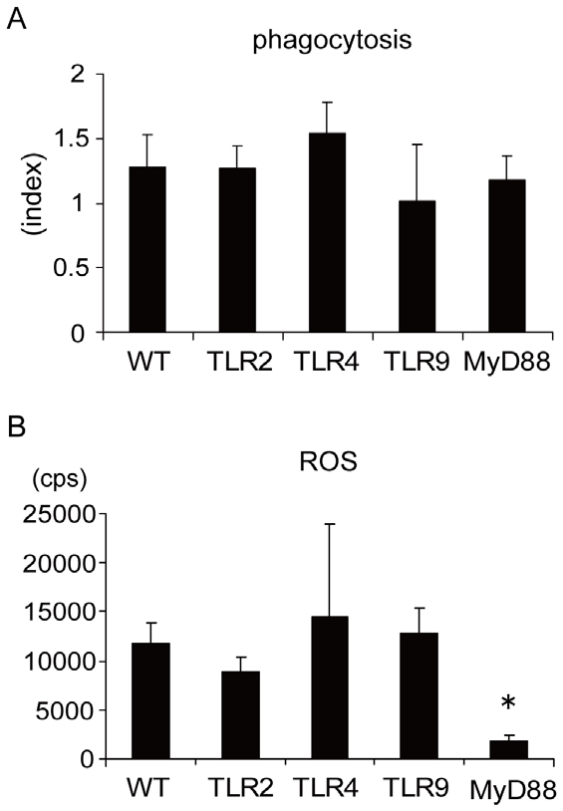
MyD88-deficient macrophage shows impaired ROS production. (A) Peritoneal macrophages were prepared from wild type and TLR2-, 4-, 9- and MyD88-deficient mice. Cells were cultured with FITC-labeled KW3110 (3 µg/ml) in a chamber slide and phagocytosis indices were measured as described in Fig. 1B. Data value is the mean ± SD of three mice. (B) Macrophage from wild type and TLR2-, 4-, 9- and MyD88-deficient mice were cultured with 1 µg/ml KW3110 for 8 hrs and ROS production was measured. Data are mean ROS intensity ± SD of three mice. * *p*<0.01 (Dunnett's test).

To study the difference between MyD88-deficient macrophage and other macrophages, we analyzed phagocytosis and ROS production of macrophages from TLR2-, 4-, 9- and MyD88-deficient mice. There was no significant change (Dunnett's test) in the phagocytosis index, indicating that the TLR is not necessary for KW3110 recognition and internalization (Fig. 5A). ROS production, on the other hand, significantly decreased (*p*<0.01; Dunnett's test) in macrophages from MyD88-deficient mice (Fig. 5B), suggesting that the macrophages from MyD88 deficient mice have difficulty inducing ROS.

## Discussion

We show here that different LAB strains display variable potential to induce IL-12 production in macrophage (Fig. 1A, Table 1). However the mechanism by which these strains induce IL-12 is still unclear. One key finding in this study is the role of ROS in LAB induced IL-12 production. It is well known that ROS are induced by macrophages following phagocytosis of microbes [31], [32] and we have shown a clear correlation between phagocytosis and both IL-12 and ROS induction in 3 LAB strains (KW3110, ATCC 53103 and NRIC1942) and that this phenomenon seems to be a general feature of LAB (Fig. 1C, D, Table 1). In addition, ROS was crucial for IL-12 production from KW3110 stimulated macrophages (Fig. 3). These results demonstrate the existence of ROS dependent IL-12 induction mechanisms in LAB stimulated macrophage. Correspondingly, ROS production was followed by IL-12 mRNA expression (Fig. 2). However, it is important to note that ROS are not always sufficient for IL-12 induction since there were some LAB strains that induced large levels of ROS but low levels of IL-12 (Table 1).

There is still much debate about how redox conditions affect IL-12 production. In some reports N-acetyl-cysteine (NAC), a potent ROS scavenger and reducing agent, inhibited IL-12 secretion from macrophages [33], [38]. Our results support these findings because ROS may induce an oxidative state in LAB stimulated macrophage. On the other hand, Utsugi et al. and Murata et al. report that reductive macrophages with a high intracellular glutathione concentration show increased IL-12 production and that oxidative macrophages with low glutathione levels show decreased amounts of IL-12 [39], [40]. This conflict might due to the different methods of macrophage stimulation. Utsugi and Murata used IFN-γ or TLR ligands for macrophage stimulation, instead of LAB, which are initially phagocytosed by macrophage. Indeed, ROS inhibition did not affect IL-12p40 secretion from CpG stimulated macrophages, suggesting that ROS is not essential for CpG mediated IL-12 induction (Fig. 3A). There might also be differences in the IL-12 induction pathway in intact LAB and individual TLR ligands.

It is reasonable to conclude that activated NADPH oxidase produces ROS after LAB phagocytosis (Fig. 1), but the ROS production is slower than previous report, in which the production peaks at 20–30 min after micro-beads phagocytosis. Different from the micro-beads [41], LAB phagocytosis is observed 4 hrs after incubation and the phagocytosis index keeps increasing for 16 hrs (not shown). This slow phagocytosis might be the reason of delayed ROS production. Recent study showed that TLR signaling leads delayed mitochondrial ROS (mROS) production and its kinetics is similar to our data [42]. Although it is interesting to test mROS system, we consider it unlikely because the ROS production is not TLR dependent as is discussed below. The signaling pathway for ROS dependent IL-12 production is still unclear. One candidate is the ASK1-mediated p38 MAPK pathway [43]–[45] in which ROS induces ASK1 phosphorylation followed by activation of the p38 MAPK cascade. It is known that the p38 MAPK-NF-κB pathway is important for IL-12 induction [46]. Indeed, our preliminary studies using the p38 specific inhibitor SB 202190 showed strongly inhibition of KW3110 induced IL-12 production (data not shown). We hope to study the ASK1-mediated p38 MAPK pathway for LAB/ROS induced IL-12 production in more detail in the future.

In many studies, IL-12 production by microbial components is TLR dependent [47]–[49]. But we have shown in a previous report that LAB induced IL-12 is MyD88 dependent, but not TLR2, 4, 9 or IL-1R, IL-18R dependent [26], a finding confirmed by this study on peritoneal macrophages (Fig. 4A). Some reports, utilizing other microbes, support our results [50]–[52]. Interestingly, the LAB strain KW3110 only had a TLR2 ligand and, despite the use of TLR2 deficient macrophage, successfully produced IL-12 (Fig. 4A, C). Also KW3110 stimulation did not induce IRAK1 degradation. We suggest that KW3110 did not induce IL-12 via a TLR signaling pathway. However, it is difficult to explain why MyD88 is necessary for IL-12 induction if KW3110 does not stimulate IL-12 via a TLR. Interestingly, MyD88 deficient macrophage phagocytosed KW3110 as frequently as did WT macrophage, suggesting that the initial LAB recognition event is totally independent of the TLR-MyD88 pathway (Fig. 5A). It is important to emphasize that LAB are not randomly phagocytosed, as demonstrated by the NRIC1942 strain, which induces poor IL-12 and is rarely detected in macrophages (Fig. 1A, B). The big difference observed in MyD88 deficient macrophages was their reduced ROS production after LAB phagocytosis (Fig. 5B). Since ROS is essential for IL-12 induction, we suggest that less ROS production is one reason for the lack of IL-12 production in MyD88 deficient macrophages. It remains unclear why MyD88 deficient mice could not induce ROS after KW3110 phagocytosis. Laroux et al. report that macrophages from MyD88 deficient mice do not produce sufficient ROS in response to *Escherichia coli* or *Salmonella typhimurium* and suggest that there are other functional disorders in the ROS inducing pathway in MyD88 deficient macrophages [53].

This is the first report on the essential role played by ROS in IL-12 production by LAB stimulated macrophage. The mechanisms by which LAB are recognized and phagocytosed remain elusive and additional studies are necessary to further our understanding of the immune modulatory functions of LAB.

## Materials and Methods

### Mice

Male C57BL/6 and BALB/c mice were purchased from Charles River Laboratories Japan (Kanagawa, Japan). Male MyD88-, TLR2-, TLR4-, TLR9-deficient mice on the C57BL/6 genetic background were obtained from Oriental Bioservice (Kyoto, Japan). Animal experiments were performed in accordance with the guidelines for care and use of laboratory animals of the Kirin Holdings Co., Ltd. The protocols were approved by the Animal Experiment Committee of the Kirin Holdings Co., Ltd.

### Materials

Synthetic diacylated lipoprotein FSL-1 was purchased from InvivoGen (San Diego, CA). LPS derived from *Salmonella typhosa* and apocynin (NADPH oxidase inhibitor) were purchased from Sigma (St Louis, MO). Oligodeoxynucleotides containing unmethylated CpG motifs (1668) were obtained from Hycult Biotechnology (Netherlands). Propyl gallate (ROS scavenger) and L-012 were purchased form Wako (Osaka, Japan) Fluorescein (FITC)-conjugated anti-mouse CD11b (clone M1/70) and allophycocyanin (APC)-conjugated anti-mouse F4/80 (clone BM8) were purchased from e-bioscience (San Diego, CA). Anti-mouse CD16/32, phycoerythrin (PE)-conjugated anti-mouse IL-12p40 (clone 15.6) and purified anti-mouse CD11b (clone M1/70) were purchased from BD PharMingen (San Diego, CA). Alexa 568-conjugated anti-rat IgG was purchased from Molecular Probes (Leiden, OR). Rabbit anti-mouse IRAK-1 polyclonal antibody and rabbit anti-actin polyclonal antibody were purchased from Santa Cruz Biotechnology (Santa Cruz, CA). Heat-killed bacterial strains used in this study, Lactobacillus rhamnosus ATCC53103, Lactobacillus paracasei NRIC1942, Lactobacillus paracasei KW3110 and others (table 1) were prepared as described previously [10].

### Preparation of Peritoneal Macrophages and Culture

Mice were injected intraperitoneally with 1 ml of 4% thioglycollate 3 days before harvesting. Peritoneal exudate cells were collected by washing the peritoneal cavity with phosphate buffered saline (PBS), and used as macrophages. Peritoneal macrophages were adjusted to 1×10∧6 cells/ml with RPMI1640 (Sigma) supplemented with 10% fetal calf serum, 100 U/ml of penicillin and 100 µg/ml of streptomycin and cultured with indicated reagents and lactobacilli.

### Flow Cytometric Analysis of IL-12 production by macrophages

Peritoneal macrophages were cultured with 1 µg/ml KW3110 strain for 20 hr. BD GolgiStop (BD PharMingen) was added 8 hr later. After culture, cells were washed and incubated with anti-CD16/32 to block Fcγ receptors followed by PE conjugated anti-CD11b and APC conjugated anti-F4/80. After washing, cells were fixed, permeabilized by Cytofix/Cytoperm kit (BD PharMingen) and stained for intracellular IL-12p40 following the manufacturer's instructions. Cells were washed and analyzed on a FACSCant2 flow cytometer (Becton Dickinson).

### Western Blotting

Peritoneal macrophages were cultured with 1 µM CpG, 1 µg/ml LPS, 1 µg/ml FSL-1 or 1 µg/ml strain KW3110. Cells were lysed on ice for 10 min in lysis buffer containing 20 mM MOPs, pH 7.0, 2 mM EGTA, 5 mM EDTA, 1% Nonidet P-40, 1 mM dithiothreitol, protease inhibitor cocktail (Roche, Switzerland) and phosphatase inhibitor cocktail (Thermo Fisher Scientific, Waltham, MA). Cell debris was pelleted by centrifugation for 20 min. The protein concentration in the supernatant was determined using Bio-Rad (Hercules, CA) protein assay kit according to the protocol provided by the manufacturer. Extracts with equal amount of proteins were solubilized by SDS sample buffer (Bio-Rad), separated by SDS-PAGE, transferred to polyvinylidene difluoride membrane (Bio-Rad), Western blotted with anti-IRAK or anti-actin antibody followed by horseradish peroxidase conjugated goat anti-rabbit IgG (Cell Signaling), and detected using the ECL Plus Western blotting detection system (GE Healthcare, UK).

### Microscopic Analysis of Phagocytosis

Peritoneal macrophages were plated on 8 well chamber slides and pre-incubated for 2 hr. FITC-labeled Lactobacilli (3 µg/ml) was added to the cell culture medium. Cells were washed using PBS and stained with anti-CD11b followed by anti-rat IgG Alexa 568 after 8 hours' culture. After staining, cells were fixed with Mildform (Wako) and observed by confocal laser scanning microscopy TCS NT (Leica Microsystems, Germany). The phagocytic index was defined in this study as the mean number of internalized bacterial cells per macrophage after incubation. A minimum of 100 macrophages from randomly scanned pictures were counted (magnification, ×630).

### Measurement of Reactive Oxygen Species and Cytokine Production

Macrophages were cultured with lactobacilli (1 µg/ml) in a 96 well white plate (200 µl) for 22 hours. L-012 was added to the cell culture medium (final concentration 500 µM) at the indicated time points and further incubated for 1 hour. Chemical luminescence counts were measured using an ARVO Light (PerkinElmer, Waltham, MA). Cell culture supernatants were harvested after 24 hours' cell culture and the amount of IL-12p40 and IL-12p70 was measured using an OptEIA ELISA kit (BD PharMingen) according to manufacturer's instructions.

### Quantitative RT-PCR

Total RNA was extracted from cultured macrophages at indicated time points by using RNAeasy mini Kit (QIAGEN) according to the manufacture's instructions. cDNA synthesis was performed with ThermoScript RT-PCR System (Invitrogen, Carlsbad, CA). The cDNA reaction mixture was added to SYBR Premix Ex Taq (Takara Bio, Otsu, Japan), containing IL-12p35 (gaccctgtgccttggtagcatc and tgcttctcccacaggaggtttc), IL-12p40 (atgcccctggagaaacagtgaa and tgtggagcagcagatgtgagtg) or GAPDH (gcctggagaaacctgccaagta and tgaagtcgcaggagacaacctg) primer pairs at 1 µM. Reactions were performed in the Light Cycler 480 (Roche), beginning with a 10 s hot-start activation of the Taq polymerase at 95°C, followed by 40 cycles of amplification in four steps (denaturation at 95°C for 5 s, annealing at 66°C for 2 s, extension at 72°C for 2 s, and fluorescence detection). The value for the target gene was divided by the value for the GAPDH gene, and relative gene expression units were normalized by the respective gene value of macrophages with no stimulation.

### TLR ligand screening

Intact KW3110 and French pressed KW3110 (10 µg/ml each) were applied for TLR ligand screening assay with HEK293 cells expressing given TLRs. The secreted alkaline phosphatase reporter is under the control of a promoter inducible by the transcription factor NF-κB. Those cells were provided by InvivoGen (San Diego, CA). And this assay was performed by InvivoGen.
